# Pulsatility of glucocorticoid hormones in pregnancy: Changes with gestation and obesity

**DOI:** 10.1111/cen.13548

**Published:** 2018-01-29

**Authors:** Laura I. Stirrat, Jamie J. Walker, Ksenia Stryjakowska, Natalie Jones, Natalie Z. M. Homer, Ruth Andrew, Jane E. Norman, Stafford L. Lightman, Rebecca M. Reynolds

**Affiliations:** ^1^ Tommy's Centre for Maternal and Fetal Health Medical Research Council Centre for Reproductive Health University of Edinburgh Edinburgh UK; ^2^ Henry Wellcome Laboratories for Integrative Neuroscience and Endocrinology University of Bristol Bristol UK; ^3^ University/BHF Centre for Cardiovascular Science University of Edinburgh Edinburgh UK; ^4^ Wellcome Trust Centre for Biomedical Modelling and Analysis University of Exeter Exeter UK; ^5^ EPSRC Centre for Predictive Modelling in Healthcare University of Exeter Exeter UK; ^6^ College of Engineering, Mathematics and Physical Sciences University of Exeter Exeter UK; ^7^ Mass Spectrometry Core Edinburgh Clinical Research Facility University of Edinburgh Edinburgh UK

**Keywords:** glucocorticoids, human pregnancy, obesity, pulsatility

## Abstract

**Objective:**

Hypothalamic‐pituitary‐adrenal axis (HPA) activity is decreased in obese pregnancy and associates with increased foetal size. Pulsatile release of glucocorticoid hormones regulates their action in target tissues. Glucocorticoids are essential for normal foetal growth, but little is known about glucocorticoid pulsatility in pregnancy. We aimed to investigate the ultradian rhythm of glucocorticoid secretion during obese and lean pregnancy and nonpregnancy.

**Design:**

Serum cortisol, cortisone, corticosterone and 11‐dehydrocorticosterone were measured by LC‐MS/MS from samples obtained at 10‐minute intervals between 08.00‐11.00 hours and 16.00‐19.00 hours, from 8 lean (BMI <25 kg/m^2^) and 7 obese (BMI > 35 kg/m^2^) pregnant women between 16‐24 weeks gestation and again at 30‐36 weeks), and nonpregnant controls (lean n = 3, obese n = 4) during the luteal phase of their menstrual cycle. Interstitial fluid cortisol was measured by ELISA, from samples obtained using a portable microdialysis and automated collection device at 20‐minute intervals over 24 hours.

**Results:**

Serum cortisol AUC, highest peak and lowest trough increased significantly with gestation in lean and obese pregnant compared with nonpregnant subjects. Pulsatility of cortisol was detected in interstitial fluid. In pregnant subjects, interstitial fluid pulse frequency was significantly lower with advancing gestation in obese, but not in lean.

**Conclusions:**

We demonstrate cortisol pulsatility in interstitial fluid. Pulse frequency is altered with increased gestation and BMI. This may be a novel mechanism to explain decreased HPA activity in obese pregnancy.

## 1 INTRODUCTION

Glucocorticoids are vital for normal foetal growth and organ development, but foetal overexposure to the major circulating glucocorticoid, cortisol, is associated with intrauterine growth restriction^1^ and an increased risk of cardiovascular disease in later life.^2^ During pregnancy, a number of endocrine changes cause the maternal hypothalamic‐pituitary‐adrenal (HPA) axis to undergo dramatic activation resulting in cortisol levels that are around threefold higher than in nonpregnancy.^3^, ^4^, ^5^ Dysregulation of the maternal HPA axis has been implicated in pregnancy complications including preterm birth^6^ and preeclampsia.^7^, ^8^ The glucocorticoid corticosterone, which is known to circulate at significantly lower levels than cortisol in nonpregnancy,^9^ is increasingly being recognized as a key player in HPA axis regulation.^10^ It has been suggested that the foetus preferentially secretes corticosterone over cortisol in response to stress, and therefore has been proposed to be an important biomarker of foetal stress at the time of delivery,^11^, ^12^ but little is known about how it changes during pregnancy.

The circadian rhythm of the HPA axis, which in humans is characterized by peak levels of cortisol early in the morning, is maintained in pregnancy.^13^ More detailed studies of circadian rhythmicity in animals^14^ and in nonpregnant humans^15^ have identified an underlying “ultradian rhythm”^16^ of cortisol pulses within blood and target tissues,^17^ occurring approximately once per hour.^16^, ^18^, ^19^ Pulse amplitude and frequency increase during the circadian peak of secretion when circulating cortisol levels are at their highest^16^ and in response to food.^20^ These pulses are important for optimal gene transcription and metabolic functions.^21^ Altered ultradian rhythm patterns have been linked to pathological consequences and manifestations of disease^21^ such as psychotic and depressive states,^22^, ^23^ where elevated troughs at the nadir result in a flattened circadian rhythmicity. In pregnancy, excess glucocorticoid exposure is thought to induce a long‐lasting effect on peripheral tissue expression of glucocorticoid‐sensitive genes.^24^ However, the underlying mechanisms for such changes are unknown. One study of cortisol pulsatility in pregnancy used 30‐minute serum sampling and detected 2‐3 pulses of cortisol in 12 hours in the third trimester of human pregnancy,^25^ but the authors acknowledged that 30‐minute sampling was suboptimal for accurate detection of pulse rates of hormones with rapid clearance rates. Whether or not and how the ultradian rhythm of cortisol secretion changes across gestation is unknown. This information will improve understanding of pathways to foetal growth as well as the aetiology of pregnancy complications.

We aimed to determine whether there are changes in cortisol pulsatility in 2 physiological contexts of altered HPA axis activity; firstly, the increase in circulating cortisol levels that occur with advancing gestation, and secondly, in maternal obesity, where we and others have reported lower maternal circulating cortisol levels in obese than in lean pregnant women.^26^, ^27^ We hypothesized that pulse amplitude and frequency would increase with advancing gestation and that these characteristics would be lower in obese compared with lean pregnant women. To test this hypothesis, we studied daytime serum profiles of both cortisol and corticosterone as well as their inactive metabolites (cortisone and 11‐dehydrocorticosterone, respectively), and 24‐hour interstitial fluid cortisol levels in obese and lean pregnant, and nonpregnant women. We conducted more frequent sampling than has previously been performed^25^ to maximize the likelihood of detecting cortisol pulses.

## 2 SUBJECTS AND METHODS

### 2.1 Subjects and clinical protocol

We recruited lean (BMI 18.5‐24.9 kg/m^2^) pregnant (LP; n = 8), obese (BMI ≥ 30 kg/m^2^) pregnant (OP; n = 7), lean nonpregnant (LNP; n = 3) and obese nonpregnant (ONP; n = 4) women. One obese participant was studied in nonpregnancy and again during pregnancy. Pregnant women were recruited from antenatal clinics in NHS Lothian. Nonpregnant volunteers were recruited from the University of Edinburgh, and community weight loss clinics in NHS Lothian. Eligible pregnant women were Caucasian and had a normal booking ultrasound scan and a singleton pregnancy. Nonpregnant women had a regular menstrual cycle and did not use hormonal contraception. Exclusion criteria were smoking, pre‐existing diabetes, regular glucocorticoid medication, severe mental health disorder and anaemia. Ethical approval and written informed consent were obtained.

Participants attended study visits at the Edinburgh Clinical Research Facility, Royal Infirmary of Edinburgh. Pregnant women attended for study visits at 16‐24 weeks’ gestation and between 30‐36 weeks’ gestation, and nonpregnant volunteers attended for one study visit in the luteal phase of the menstrual cycle.

At each study visit, fasting blood samples were obtained at 10‐minute intervals between 08.00‐11.00 hours and between 16.00‐19.00 hours via a peripheral venous cannula. Subcutaneous interstitial fluid samples for measurement of free cortisol^28^ were obtained at 20‐minute intervals over 24 hours by microdialysis. A linear microdialysis catheter (Linton, Norfolk, UK) was inserted subcutaneously into the interstitial compartment of the anterior abdominal wall and collected via a novel, miniaturized, portable collection device as described by Bhake et al^15^

Serum was separated immediately and stored at −80°C for later laboratory analysis. Clinical outcomes including maternal booking BMI, gestation at delivery, birthweight and offspring gender were extracted from medical records.

### 2.2 Laboratory methods

#### 2.2.1 Liquid chromatography‐tandem mass spectrometry (LC‐MS/MS)

We measured cortisol, cortisone, corticosterone and 11‐dehydrocorticosterone simultaneously by liquid chromatography‐tandem mass spectrometry, using a Waters Acquity™ UPLC (Manchester, UK), liquid chromatography system followed by mass spectral analysis on an ABSciex QTRAP^®^ 5500 (Warrington, UK) mass spectrometer. Mass spectral conditions are described in Table [Link], [Link], [Link], [Link] in conjunction with ion spray voltage (5500 V) and source temperature (700°C). Following enrichment of serum (200 μl) with internal standards, epi‐cortisol (25 ng; Steraloids, USA), epi‐corticosterone (25 ng; Steraloids, USA) and 9,12,12,12 [^2^H_4_]‐cortisol (D4‐cortisol 25 ng; QMX Laboratories, England, UK), and dilution with water (200 μl) analytes were extracted via supported liquid extraction (ISOLUTE^®^ SLE+ 400 μl 96‐well plate, Biotage, Sweden) and eluted with 98:2 dichloromethane:2‐propanol. Analytes were separated on an ACE Excel 2 C18‐AR (150 × 2.1 mm, 2 μm) column (Advanced Chromatography Technologies Ltd, UK) at 40 °C. The elution process started with 70:30 water with 0.1% formic acid (FA) (solution A) and acetonitrile with 0.1% FA (solution B) was maintained for 4 minutes followed by a 1‐minute linear rise to 60% solution B, a subsequent rise to 90% solution B, before returning to 30% solution B by 6.1 minutes at a constant flow rate of 0.5 mL/min.

Validation parameters (intra‐ and interassay precision, accuracy; Table S2) were within acceptable limits (the lowest levels being acceptable <20% RSD with all following <15%). Levels of corticosterone and 11‐dehydrocorticosterone in some samples were close to or below the limit of quantification in all subjects and at all gestations of pregnancy. The ratios of cortisol: cortisone and corticosterone: 11‐dehydrocorticosterone were used as a marker of 11beta‐hydroxysteroid dehydrogenase type 2 (11β‐HSD2) enzyme activity.

Commercially available ELISA kits were used for the analysis of plasma ACTH (Demeditec DE3467; interassay CV 6.9‐7.1%) and interstitial fluid cortisol (IBL International RE52611; interassay CV 4.2‐17.0%), as previously used by Bhake et al^15^ Free cortisol values were expected to be above the top standard, therefore were diluted to 1:10 in the assay zero standard and results were corrected for dilution.

### 2.3 Statistical analysis

Data distribution was assessed for normality by visually assessing histograms. Data that were not normally distributed were normalized using the natural‐log transformation. The independent *t* test was used to test for differences in subject characteristics between continuous variables and chi‐squared test for categorical variables. For cortisol measurements, the “highest peak” was the highest value in any given profile, and the “lowest trough” was the lowest value. The area under the curve (AUC) for hormone profiles was used as a marker for total glucocorticoid levels over the study period. Pulse analysis was performed using Cluster analysis,^29^ a statistically rigorous peak detection algorithm that has been widely used to quantify the pulsatile dynamics of various hormones, including cortisol.^30^ The algorithm detected statistically significant interstitial fluid cortisol pulses, pulse frequency (pulses/hours), pulse height (μg/dl) and mean concentration (μg/dl) in a given cortisol time series. Cluster parameters used in the analysis were as follows: minimum detectable concentration of the assay (MDC, 0.015 μg/dl); intra‐assay coefficient of variation (CV, 12.08%); test cluster size for sliding nadir (2.0); test cluster size for sliding peak (1); *t*‐statistic for significant increase in the data (2.0); *t*‐statistic for significant decrease in the data (2.0); and minimum peak size (0.0 μg/dl). One‐way ANOVA was used to compare changes in pulse characteristics between different groups. The paired *t* test was used to assess differences in pulse characteristics between pregnant women who attended for 2 study visits. Analysis was performed using SPSS v21 (IBM). Data in text are mean ± (SD), and data in figures are mean ± SEM. Statistical significance was considered at *P* < .05.

## 3 RESULTS

### 3.1 Demographics

Maternal and neonatal characteristics are demonstrated in Table 1. In pregnant participants, OP were younger and had higher systolic and diastolic blood pressure than LP. The timing of study visit 1 tended to be earlier in LP, and gestation at delivery was earlier in OP. There were no differences in parity, birthweight percentile or standard deviation birthweight score. In nonpregnant participants, obese subjects were older and also had higher systolic and diastolic blood pressure than lean.

**Table 1:** Maternal and neonatal characteristics

		Nonpregnant			Pregnant		
		Lean (n = 3)	Obese (n = 4)	*P*‐value	Lean (n = 8)	Obese (n = 7)	*P*‐value
Demographics	Age (years)	36.3 (4.2)	39.0 (4.1)	.435	35.1 (1.7)	29.3 (5.3)	.026*
	BMI (kg/m^2^)	22.3 (2.1)	36.5 (3.3)	.001*	21.9 (1.6)	43.7 (5.3)	<.0001*
	Weight (kg)	66.8 (8.9)	101.2 (11.1)	.006*	60.5 (7.1)	128.3 (16.6)	<.0001*
	Blood Pressure						
	Systolic (mmHg)	112 (8)	127 (9)	.086	112 (8)	125 (9)	.011*
	Diastolic (mmHg)	70 (5.6)	79 (7.3)	.048*	66 (6)	73 (8)	.063
	Parity N (%)						
	Primiparous	N/A	N/A	N/A	4 (50)	3 (42.8)	.736
	Para 1				3 (37.5)	2 (28.1)	
	Para 2				1 (12.5)	2 (28.1)	
	Gestational age at study visit (days)	N/A	N/A	N/A			
	Pregnancy visit 1				132 (7.9)	147 (14.4)	.03*
	Pregnancy visit 2				228 (9.3)	235 (17.9)	.39
Offspring Characteristics	Gestational at delivery (days)	N/A	N/A	N/A	285 (6.6)	266 (12.9)	.003*
	Offspring gender						
	Male	N/A		N/A	5	0	.01*
	Female		N/A		3	7	
	Infant size						
	Birthweight (g)	N/A	N/A	N/A	3756 (82.8)	3283 (343.7)	.01*
	Birthweight centile				61.5 (14.0)	65.0 (25.4)	.74
	SDS Score				0.31 (0.4)	0.56 (0.9)	.52

Age, BMI, weight and blood pressure were recorded at “booking” in pregnant subjects and study visit 1 in nonpregnant subjects. Parity was defined as “primiparous” (no previous pregnancies delivered after 24 weeks), “Para 1” (one previous delivery at 24 weeks) and “Para 2” (2 or more previous deliveries after 24 weeks gestation). Birthweight excludes one baby of an obese subject, who was born preterm at 34 weeks gestation. Data are mean (SD) or N (%). Key: BMI, body mass index; SDS, standard deviation birthweight score; N/A, not applicable. Significant figure is represented by asterisk (*).

### 3.2 Serum hormone profiles (08.00‐11.00 hours and 16.00‐19.00 hours)

**Figure 1:**
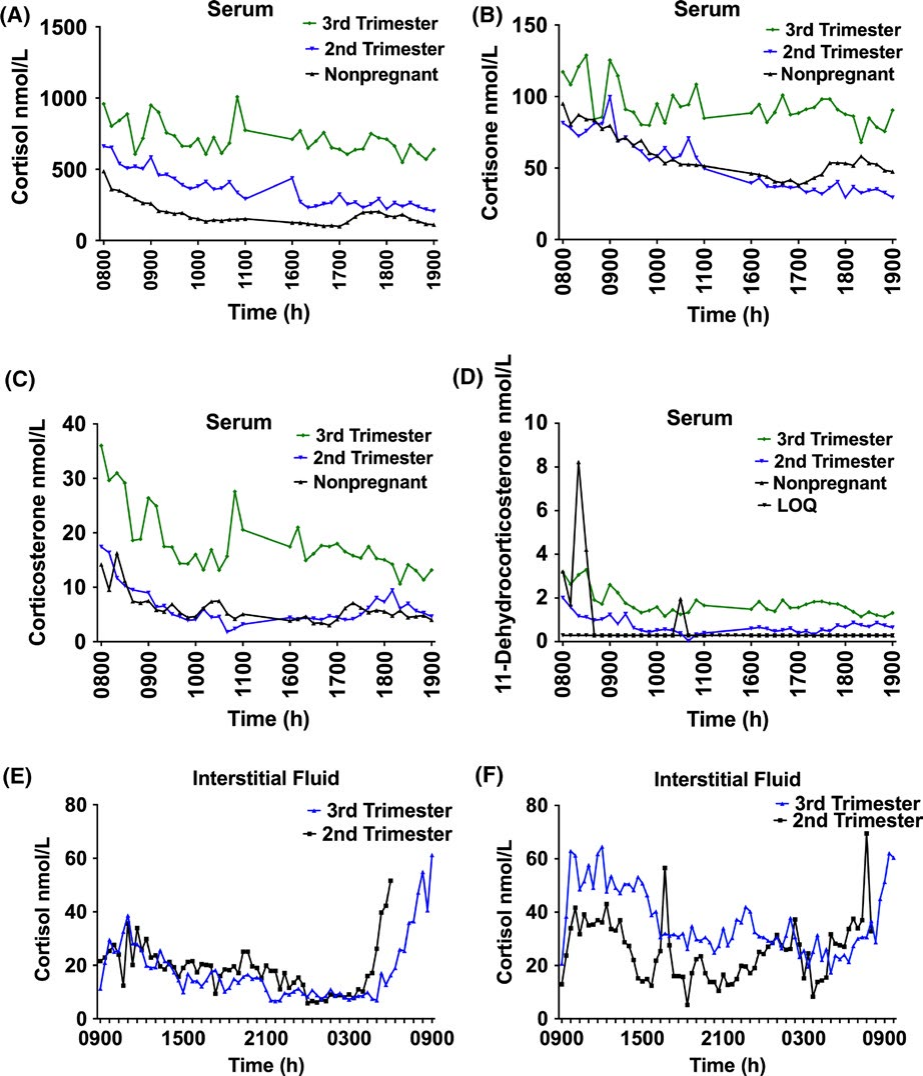
A‐F, Serum and interstitial fluid. Profiles of serum cortisol (A), cortisone (B), corticosterone (C) and 11‐dehydrocorticosterone (D). Each figure demonstrates the individual hormone profiles of one representative subject who was studied 2 months prepregnancy, at visit 1 (17 + 2 weeks’ gestation) and visit 2 (third trimester (30 + 6 weeks’ gestation). Interstitial fluid cortisol profiles from 2 representative lean subjects obtained at 17 + 2 and 31 weeks (E) and 18 + 3 and 32 + 3 weeks (F). Key: LOQ (limit of quantification) [Colour figure can be viewed at http://wileyonlinelibrary.com]

Figure 1 shows an example of serum glucocorticoid hormone profiles in nonpregnancy and during the 2 pregnancy visits, as illustrated by the data collected from the obese subject who was studied in nonpregnancy (2 months prepregnancy), and during pregnancy at visit 1 (17 + 2 weeks’ gestation) and visit 2 (30 + 6 weeks’ gestation). Levels of cortisol (Figure 1A), cortisone (Figure 1B), corticosterone (Figure 1C) and 11‐dehydrocorticosterone (Figure 1D) were higher in pregnancy than in nonpregnancy, and all hormone levels increased with advancing gestation. Profiles of serum cortisol, cortisone, corticosterone and 11‐dehydrocorticosterone for all other study participants are shown in Fig. [Link], [Link], [Link], [Link]. There were no samples from some subjects for the following reasons: did not attend visit 2 (LP2 n = 1 and OP2 n = 2) and delivered before visit 2 (OP2 n = 1). With visual assessment of hormone profiles, we observed interindividual variation in pattern of profiles, but there were no obvious differences in characteristics between women that had particularly quiescent or dynamic profiles. Only one cortisol pulse was detected in the sampling from one subject so serum pulse characteristics are not reported. Interstitial fluid pulses were detected in the profiles of all subjects. Pulse characteristics are demonstrated in Table S3, and key findings are described below.

### 3.3 Serum cortisol and cortisone

**Figure 2:**
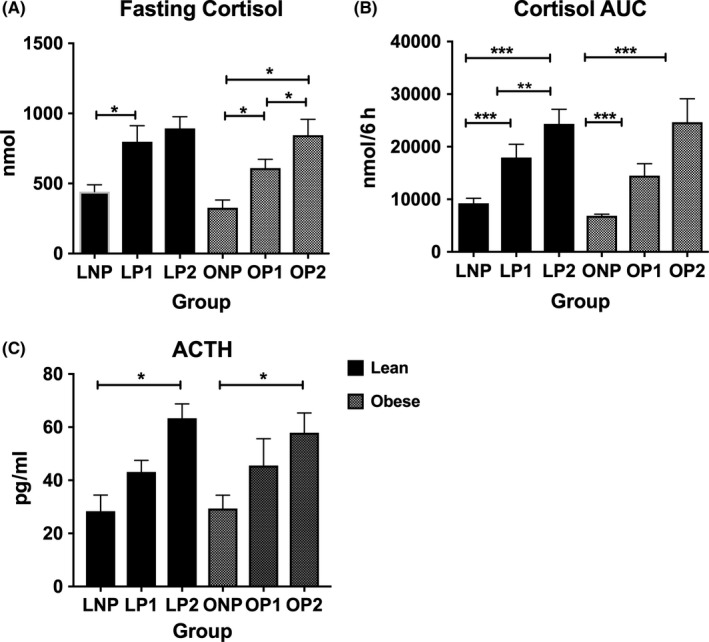
(A‐C) Fasting serum cortisol (A) increased with advancing gestation in lean and obese (one‐way ANOVA: lean *P* = .017, obese *P* = .027) and was significantly higher at OP2 than OP1 (paired *t* test *P* = .012) and at OP2 than ONP (post hoc Tukey, *P* = .046). Cortisol AUC of serum profile (B) increased with advancing gestation in lean and obese (one‐way ANOVA: lean *P* < .0001, obese *P* < .0001). AUC increased significantly between visit 1 and visit 2 in lean pregnant women (LP1 17937 ± 7141 nM vs LP2 2345 ± 7328 nM, *P* = .001), but not in obese (OP1 14498 ± 5794 vs OP2 24660 ± 8937 nM, *P* = .06). Plasma ACTH levels (C) increased during pregnancy and were significantly higher at pregnancy visit 2 than nonpregnancy in lean (*P* = .017) and obese (*P* = .045). Key: Lean nonpregnant (LNP), lean pregnant visit 1 (LP1), lean pregnant visit 2 (LP2), Obese nonpregnant (ONP), obese pregnant visit 1 (OP1), obese pregnant visit 2 (OP2). Data on graphs are mean (SEM). *P < .05, **P < .01, ***P < .0001

Fasting serum cortisol and cortisol AUC were significantly higher in pregnancy than nonpregnancy in lean and obese (Figure 2A,B). During pregnancy, cortisol AUC increased significantly with advancing gestation in lean, but not in obese (Figure 2A,B). Plasma ACTH rose with advancing gestation in both lean and obese groups (Figure 2C). The mean “highest peak” and “lowest trough” increased significantly with gestation in both lean and obese (Table S3).

Serum cortisone AUC was higher in pregnancy than nonpregnancy in both lean and obese. During pregnancy, overall cortisone levels rose (Figure 1B). Cortisone “lowest trough” increased significantly during pregnancy in obese, but not in lean (Table S3).

The mean cortisol:cortisone ratio of serum profiles was highest at pregnancy visit 1 (Table 2). The ratio of the baseline (08.00 hours) sample was significantly higher in pregnancy than nonpregnancy in obese but not in lean.

**Table 2:** Cortisol:Cortisone ratio

	Lean					Obese				
	LNP	LP1	LP2	*P* ^1^	*P* ^2^	ONP	OP1	OP2	*P* ^1^	*P* ^2^
Cortisol: Cortisone Baseline sample	4.6 (1.1)	7.9 (4.2)	6.9 (3.3)	.22	.002*	4.3 (0.7)	6.7 (1.1)	5.7 (1.9)	.007	.018*
Cortisol: Cortisone Mean overall	3.3 (0.4)	5.8 (3.1)	5.6 (2.1)	.135	.004*	3.5 (0.4)	5.9 (0.9)	5.3 (1.8)	.001	.078
Corticosterone: 11‐dehydrocorticosterone Baseline sample	2.5 (1.1)	6.4 (5.6)	5.9 (3.8)	.439	.01*	8.0 (10.3)	4.2 (3.0)	4.8 (2.5)	.728	.129

Cortisol: cortisone ratios of the first baseline fasting sample, mean of all samples, mean of first sampling time (08.00‐11.00 hours) and mean of second sampling time (16.00‐19.00 hours). One‐way ANOVA found that cortisol:cortisone on the first baseline sample and throughout the profile was significantly higher in pregnancy than nonpregnancy in obese but not in lean. The paired *t* test showed that during pregnancy, fasting cortisol:cortisone was significantly higher at visit 1 than visit 2 in lean and in obese and that cortisol:cortisone ratio for the whole profile was significantly higher in lean but not in obese. Corticosterone: 11‐dehydrocorticosterone was significantly higher at pregnancy visit 1 than visit 2 in lean, but not in obese. The ratio of corticosterone: 11‐dehydrocorticosterone was not assessed in whole profiles, as a higher proportion of samples were close to or below the limits of quantification. Data are mean (SD). One‐way ANOVA *P*‐value (*P* ^1^). Paired *t* test of pregnancy visit 1 and visit 2 (*P* ^2^). Key: LNP, lean nonpregnant; LP1 lean pregnant visit 1; LP2, lean pregnant visit 2; ONP, obese nonpregnant; OP1, obese pregnant visit 1; OP2, obese pregnant visit 2. Significant figure is represented by asterisk (*).

### 3.4 Serum corticosterone and 11‐dehydrocorticosterone

Serum corticosterone levels were highest during pregnancy visit 2 (Figure 1C) and “highest peak” increased significantly in obese during pregnancy (Table S3). Serum 11‐dehydrocorticosterone levels were not significantly different with changing gestation (Figure 1D) and did not differ between obese and lean. The ratio of corticosterone:11‐dehydrocorticosterone of the baseline fasting sample was significantly higher at LP1 than LP2, but was not different in obese (Table 2).

### 3.5 Interstitial fluid cortisol

Interstitial fluid data were available from LNP (n = 3), LP1 (n = 7), LP2 (n = 6), ONP (n = 4), OP1 (n = 7) and OP2 (n = 3). There were no samples available from some subjects for the following reasons: technical issues (LP1 n = 1 and OP2 n = 1), participant declined (LP2 n = 1), did not attend for visit 2 (LP2 n = 1 and OP2 n = 2) and delivered before visit 2 (OP2 n = 1). Representative profiles from 2 subjects are shown in Figure 1E‐F. Both pulsatility and the morning rise in cortisol were detected in the interstitial fluid sampling (Figure 1E‐F). Pulses were detected in interstitial fluid samples. In lean women, pulse frequency was consistently around 0.2 pulse/hours (approximately one pulse every 5 hours) and did not differ between lean nonpregnant and pregnant women at visit 1 and visit 2 (*P* = .591). In obese women, pulse frequency was similar to lean in nonpregnancy (0.22 ± 0.12 pulse/hours). Pulse frequency was significantly lower with advancing gestation in obese women (visit 1: 0.18 ± 0.12 pulse/hours, visit 2: 0.04 ± 0.003 pulse/hours; *t* test *P* = .025). There were no differences in pulse amplitude or mean concentration of the whole profiles with increasing gestation, or between lean and obese.

## 4 DISCUSSION

Using 2 paradigms of altered HPA axis activity in pregnancy to investigate ultradian rhythms of glucocorticoid hormone secretion, we have demonstrated changes in serum glucocorticoid levels that occur across gestation and in pregnancies complicated by obesity. Total circulating serum cortisol levels were higher in pregnancy than nonpregnancy in lean and obese and increased significantly with advancing gestation in lean but not in obese. In addition, through measurement of interstitial fluid cortisol levels, we show evidence, for the first time, of tissue cortisol pulsatility in human pregnancy. In obese pregnancy, interstitial fluid pulse frequency was lower with advancing gestation.

The observation that serum cortisol AUC significantly increased during pregnancy in lean women, but not in obese women adds to the previous observations of lower morning fasting cortisol in obese^26^, ^27^ and supports the hypothesis that HPA axis activity is reduced throughout the day in obese pregnancy.^26^ These data, together with our finding that interstitial fluid pulse frequency was lower with advancing gestation in obese pregnant women, contribute to our understanding of the changes in the HPA axis in obese pregnancy. Altered ultradian rhythm (characterized by reduced interstitial fluid pulse frequency) may be cause or consequence of decreased HPA axis in obese pregnancy. The drivers to the altered ultradian rhythm are poorly understood. With rodent and sheep data demonstrating a mismatch in timing of CRH and cortisol pulses suggesting a subhypothalamic origin, it is hypothesized that interactions between central negative feedback at the pituitary and a delay in feedforward of ACTH on adrenal production of glucocorticoids drive ultradian rhythmicity.^21^ In the absence of repeated ACTH sampling, it is not possible to determine the influence of adrenal delay in cortisol release or altered central negative feedback on pulse frequency. Whether placental production of CRH contributes to the observed alteration in ultradian rhythm has not been studied.

To the best of our knowledge, we are the first to describe circulating corticosterone levels across the day in human pregnancy and in nonpregnant women. Our finding of higher levels of corticosterone in pregnancy than nonpregnancy suggests that like cortisol, the synthesis and release of this hormone are influenced by increased activation of the maternal HPA axis.^3^ Unlike cortisone, levels of 11‐dehydrocorticosterone did not change significantly with advancing gestation, suggesting that there may be a lesser breakdown of corticosterone than cortisol by 11β‐HSD2 or alternative pathways of corticosterone clearance. For example, the ABC‐transporters “p‐glycoprotein” (P‐gp, encoded by *ABCB1*) and “multidrug‐resistant protein‐1″ (MRP1, encoded by *ABCC1*) which preferentially export cortisol or corticosterone, respectively, are present in the placenta,^31^ and *ABCB1* mRNA expression is reported to be lower in placentas of pregnancies complicated by severe obesity.^32^ Further studies are needed to understand the metabolism of corticosterone in pregnancy.

Our study is the first to measure interstitial fluid cortisol in women and in human pregnancy. We analysed the pulsatile characteristics of this data using the well‐established Cluster algorithm.^29^ This is a statistically rigorous method that takes into account assay precision and is not adversely influenced by drifting baseline hormone secretion. Moreover, its model‐independent nature means that it is not reliant on a priori assumptions about the system (eg parameters defined dynamics of hormone secretion or clearance) that we have little information about lean or obese pregnancy conditions. Using this algorithm, we demonstrated tissue cortisol pulsatility in women and in human pregnancy.

Studies of glucocorticoid pulsatility in pregnancy are limited, and to the best of our knowledge, there are no studies measuring both circulating and tissue interstitial fluid cortisol pulsatility in humans to which our data can be directly compared. Although the sample type, sampling frequency, gestation of pregnancy and lack of control group mean that our data cannot be directly compared, it is interesting to note that the interstitial fluid cortisol pulse frequency in our data was similar to the findings of a previous study of circulating serum cortisol in pregnancy. Magiakou et al^25^ used the detect pulse analysis method^33^ to identify cortisol pulses in samples obtained at 30‐minute intervals and observed 2‐3 pulses in a 12‐hour period in pregnant women between 34 and 36 weeks’ gestation. Although the authors acknowledged that the 30‐minute sampling may have limited their detection of cortisol pulses, our observations of no serum cortisol pulses over the 6‐hour sampling frame in all but one subject, and the similar interstitial fluid cortisol pulsatility to Magiakou et al, suggest that circulating cortisol pulsatility in pregnancy is decreased compared with the findings of previous studies of circulating cortisol in men^16^, ^18^ and in rats^19^ reporting that pulses occur approximately hourly.^16^, ^18^, ^19^ We sampled serum more frequently (10‐minute sampling interval) to increase the likelihood of identifying serum glucocorticoid pulses and conducted our studies with woman in the fasting state to exclude the pulsatile response that has been reported to occur in response to food.^20^ For these reasons, the sampling duration was shortened (6 hours at each visit) to limit the total blood volume sampled from pregnant women. We can only speculate that this shorter sampling time was not long enough to detect pulses using Cluster. This analytical method defines a pulse as a statistically significant increase in a “cluster” of hormone values followed by a statistically significant decrease in a second cluster of values. The increase and decrease are judged in relation to the actual experimental error expressed by replicates in the presumptive nadir and peak results. We detected significant differences in the serum cortisol highest peaks and lowest troughs of serum profiles between study groups, and thus, with a longer sampling time and a greater number of samples, these may have been recognized as statistically significant pulses.

We acknowledge that the frequency of cortisol pulses detected in interstitial fluid in lean women (approximately one pulse per 5 hours; not different between pregnant or nonpregnant women) is lower than has been reported in other studies of circulating cortisol pulse secretion^16^ in men. Rodent studies have demonstrated that circadian and ultradian rhythms of glucocorticoids are highly synchronized between the blood and subcutaneous tissue^34^ so it would be reasonable to consider that a similar frequency of cortisol pulses could be detected in interstitial fluid in our study. One possible reason for the lower pulse frequency detected in our interstitial fluid samples could be the frequency of sampling (20‐minute sampling, which allowed for continuous sampling without changing tubing or microdialysis perfusion fluid during the study). However, in the absence of a longer period of serum sampling and identification of serum pulses, it is not possible to predict whether or not the reduction in pulse frequency we detected is physiologically different from the approximately hourly secretion of cortisol pulses that has been reported in men.

Studies of the dynamic nature of glucocorticoids in humans are challenging, due to the need to obtain frequent samples. This is particularly pertinent in pregnant women where there may be ethical restrictions on the volume of blood sampled over the day. However, as our understanding of the role of the ultradian rhythm of cortisol in transcriptional regulation increases, and associations between an altered ultradian rhythm and disease processes are described,^21^ there is a need for dynamic studies. This is particularly so in the context of pregnancy, where the links between glucocorticoid exposure in utero and adverse foetal outcomes are well described, but mechanisms are not well understood. The implications of altered ultradian rhythm of cortisol in pregnancy are vast due to the wide‐ranging effects of glucocorticoids on foetal/placental gene expression or functions such as energy metabolism, lipolysis, lipid metabolism, gluconeogenesis and amino acid metabolism. This offers a potential underlying mechanism for the reduced foetal growth^1^ and increased risk of cardiovascular disease later in life^2^ that have been linked to increased glucocorticoid exposure. Conversely, for obese, it is plausible that the reduced maternal ultradian rhythm and downstream consequences may contribute to the observed increased foetal growth and delay in gestation at delivery in pregnancies complicated by obesity.^26^

Strengths of our study include frequent sampling, matched serum and interstitial fluid sampling, sampling at 2 different points in gestation and use of a robust statistical method for pulse detection and analysis. Obtaining serum samples via a peripheral cannula, and interstitial fluid samples via a microdialysis catheter, meant that frequent sampling could take place without repeated insertion of needles, which would mount a stress response. Another strength is that serum samples were analysed with gold‐standard analytical technique of liquid chromatography‐tandem mass spectrometry (LC‐MS/MS),^4^ which also allowed the simultaneous detection of multiple analytes from a small volume of sample.

A limitation of our study is that the serum sampling periods were relatively short (two 3‐hour study periods), during which only one cortisol pulse was detected in one subject's sampling profile. These durations were selected so that sampling could be performed with subjects fasting, to avoid the known pulsatile response to food,^20^, ^35^ and so that a comparison could be made between morning and evening cortisol levels, whilst considering blood withdrawal restrictions of pregnant women. We recognize that our small sample size is a limitation of this study. We acknowledge that mathematical algorithms for hormone pulse analysis have not been previously tested in a pregnant population, where there is an unknown contribution of the placenta to cortisol metabolism and clearance. We could not directly examine placental cortisol metabolism and transfer in this study. We used the ratio of cortisol:cortisone to infer total body 11β‐HSD2 activity, which we assume in pregnancy is largely reflective of the placenta. The relative reduction in the ratio between visit 1 and visit 2 of pregnancy suggests that the activity of 11β‐HSD2 increases at the later gestations, thus serving to protect the foetus from exposure to excessively high maternal cortisol levels during the third trimester.^3^ Further studies are needed to understand more about the contribution of the placenta to maternal and foetal glucocorticoid exposure.

To conclude, our study has demonstrated that total daytime circulating maternal cortisol increases with advancing gestation in lean, but not obese women. Further, we have demonstrated the novel finding that tissue cortisol levels are pulsatile during human pregnancy and that pulse frequency is significantly lower with advancing gestation in obese pregnancy. This may be an underlying mechanism for the reduced HPA axis activity we previously reported in obese pregnancy.^26^ A better understanding of the role of HPA axis dysregulation in adverse pregnancy outcomes may inform us which high‐risk pregnancies should be targeted to improve the health of the pregnant woman and the developing baby.

## CONFLICT OF INTEREST

The authors have nothing to declare.
