# Supplementary figures and images for: Pulsatility of glucocorticoid hormones in pregnancy: Changes with gestation and obesity
Source: Clin Endocrinol (Oxf). 2018 Jan 29;88(4):592–600. doi: 10.1111/cen.13548 (PMC5887976; doi:10.1111/cen.13548)

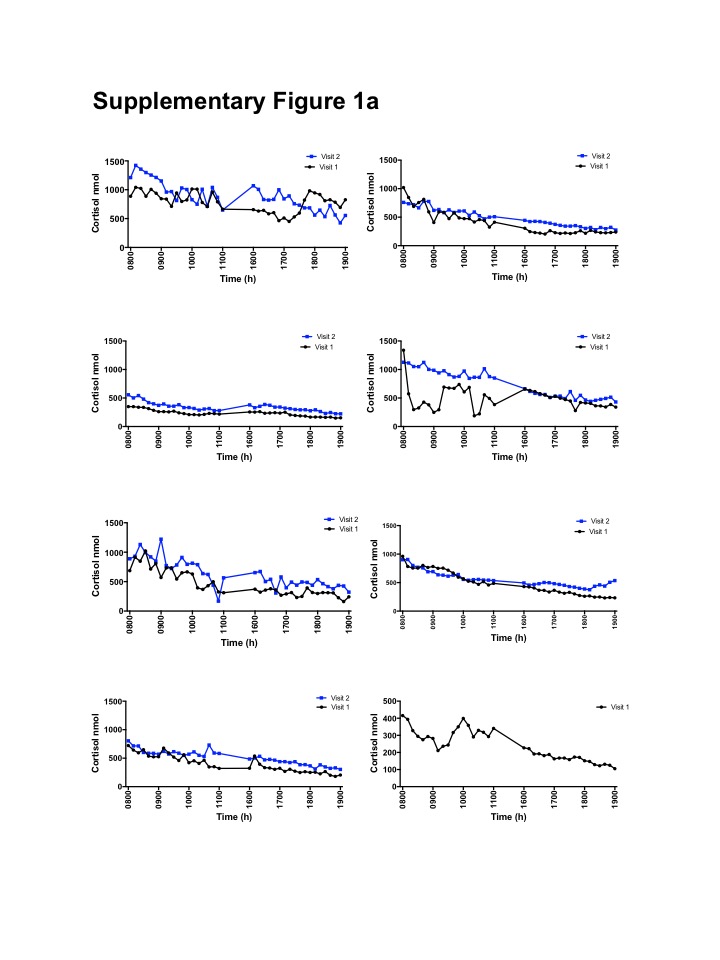

Supplement: Supplementary file 1 [file CEN-88-592-s001.jpg]

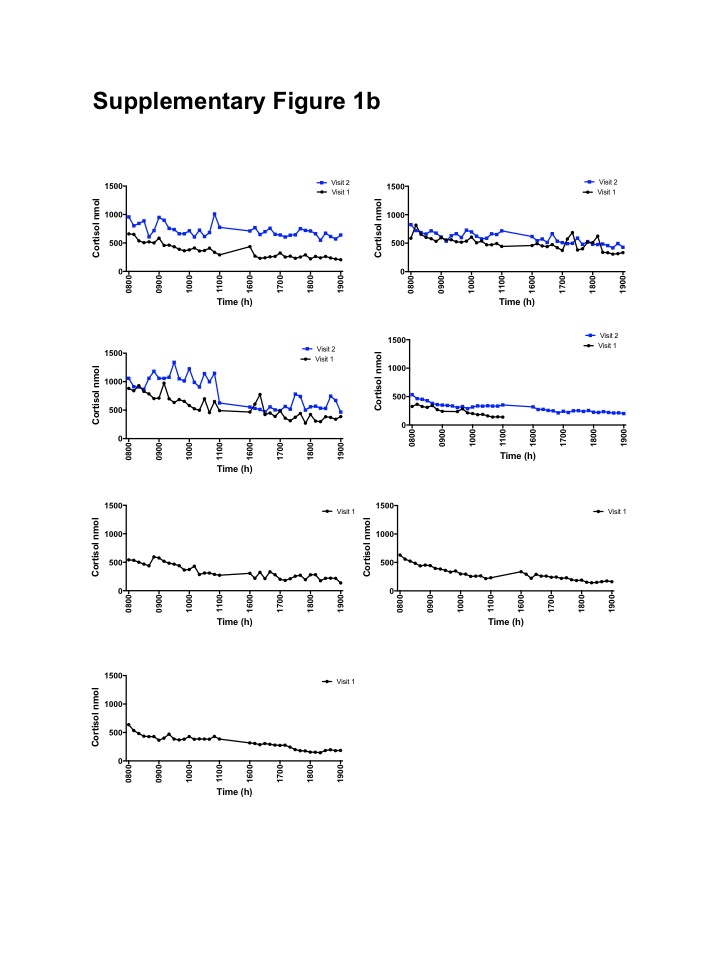

Supplement: Supplementary file 2 [file CEN-88-592-s002.jpg]

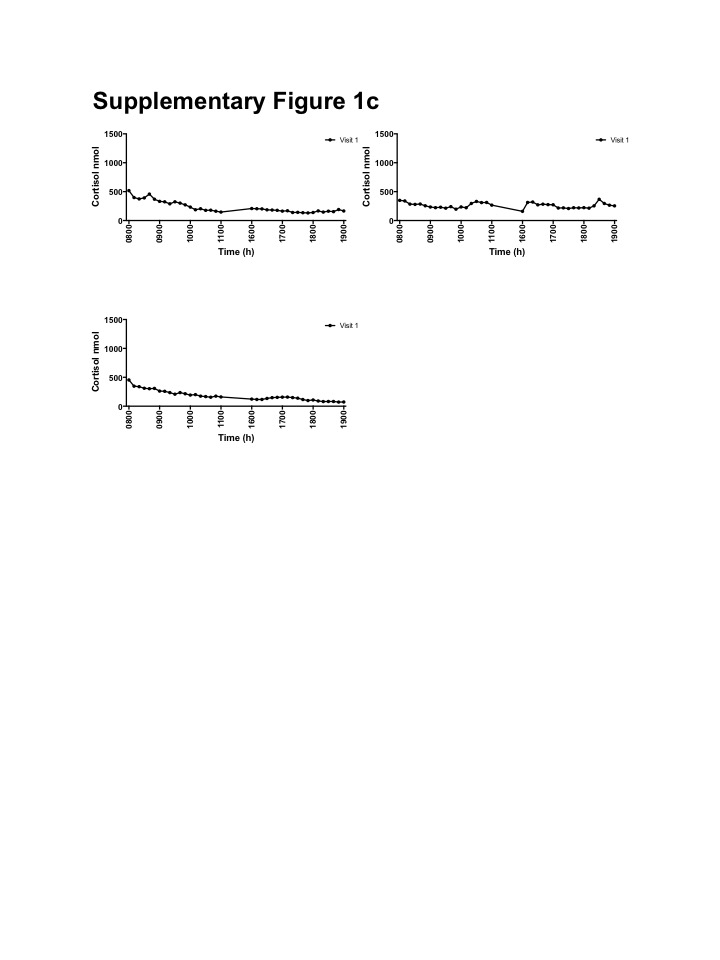

Supplement: Supplementary file 3 [file CEN-88-592-s003.jpg]

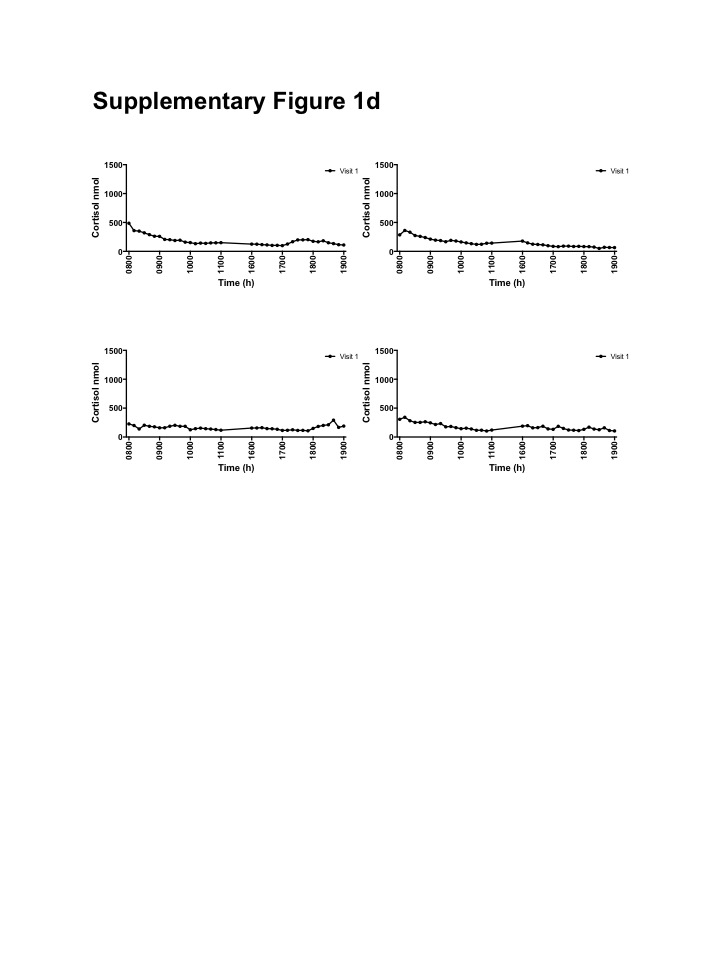

Supplement: Supplementary file 4 [file CEN-88-592-s004.jpg]

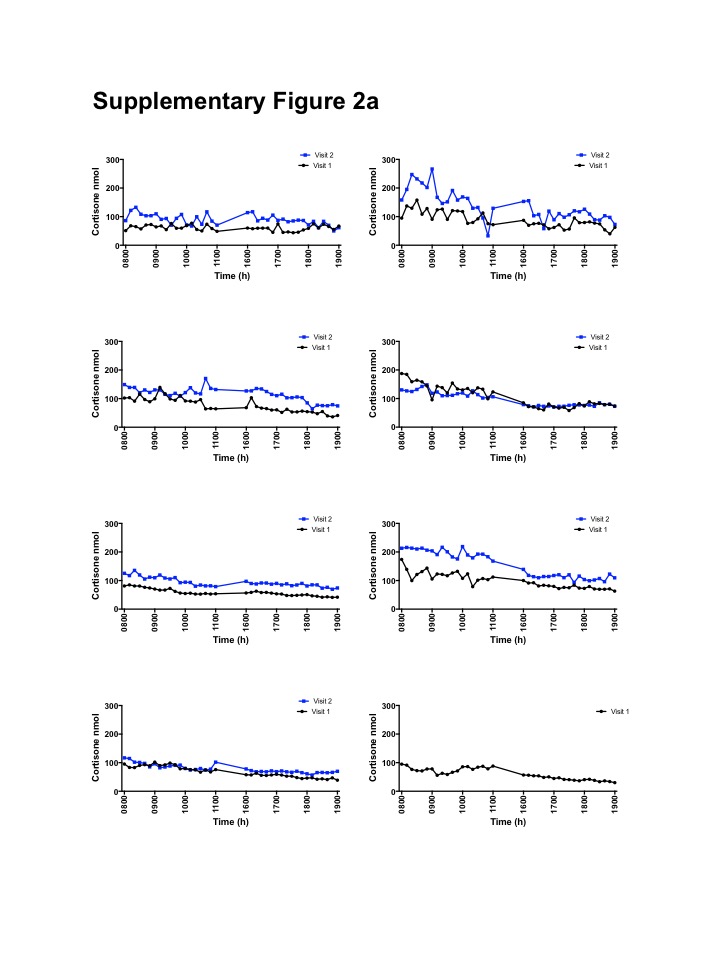

Supplement: Supplementary file 5 [file CEN-88-592-s005.jpg]

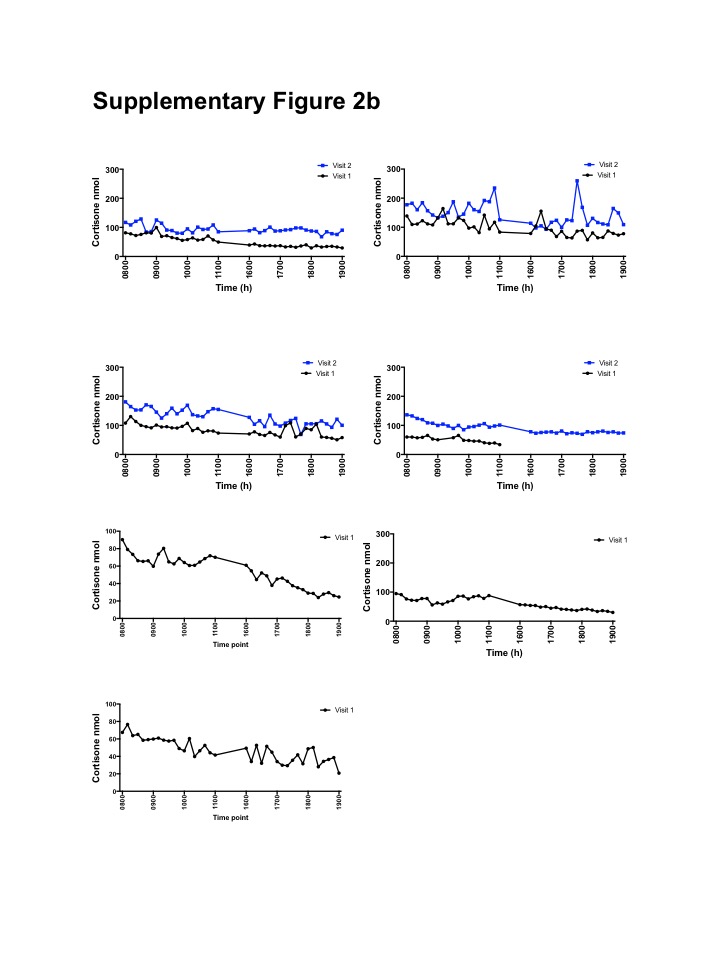

Supplement: Supplementary file 6 [file CEN-88-592-s006.jpg]

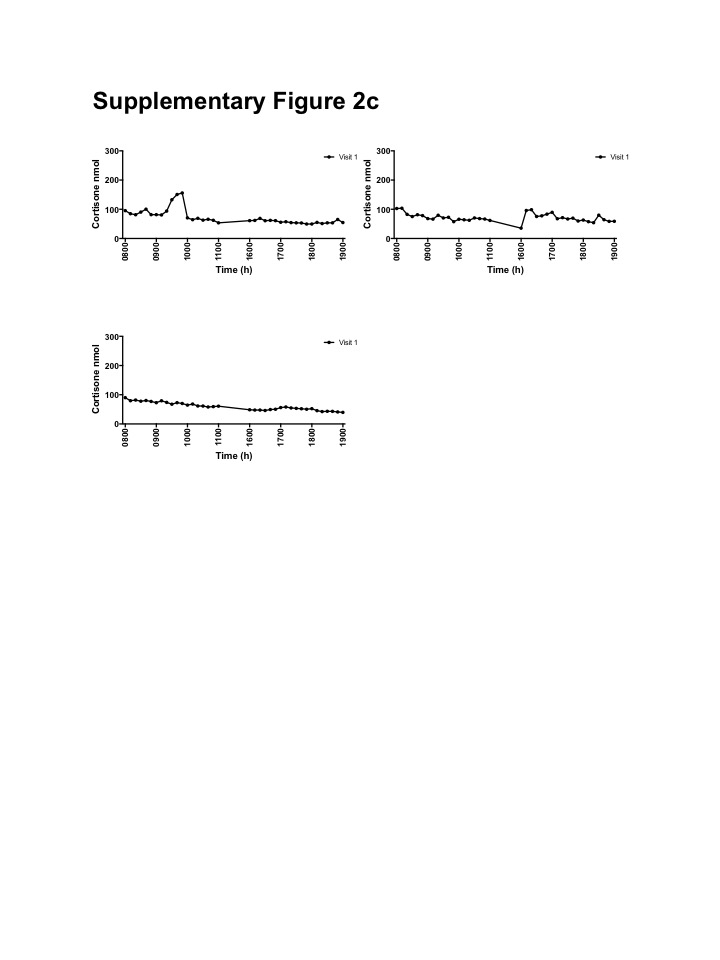

Supplement: Supplementary file 7 [file CEN-88-592-s007.jpg]

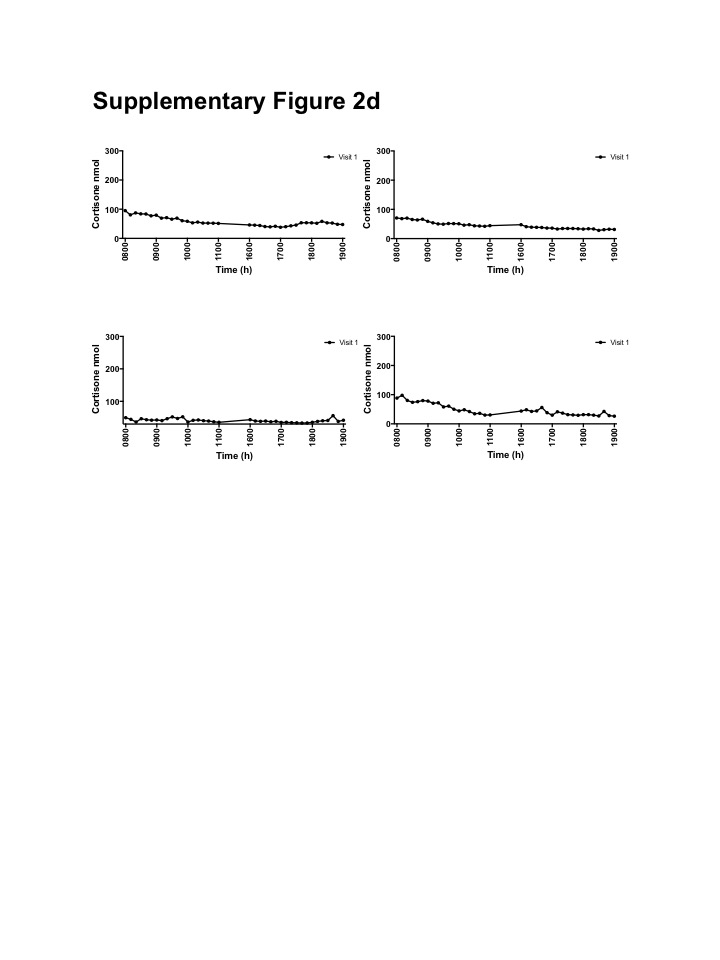

Supplement: Supplementary file 8 [file CEN-88-592-s008.jpg]

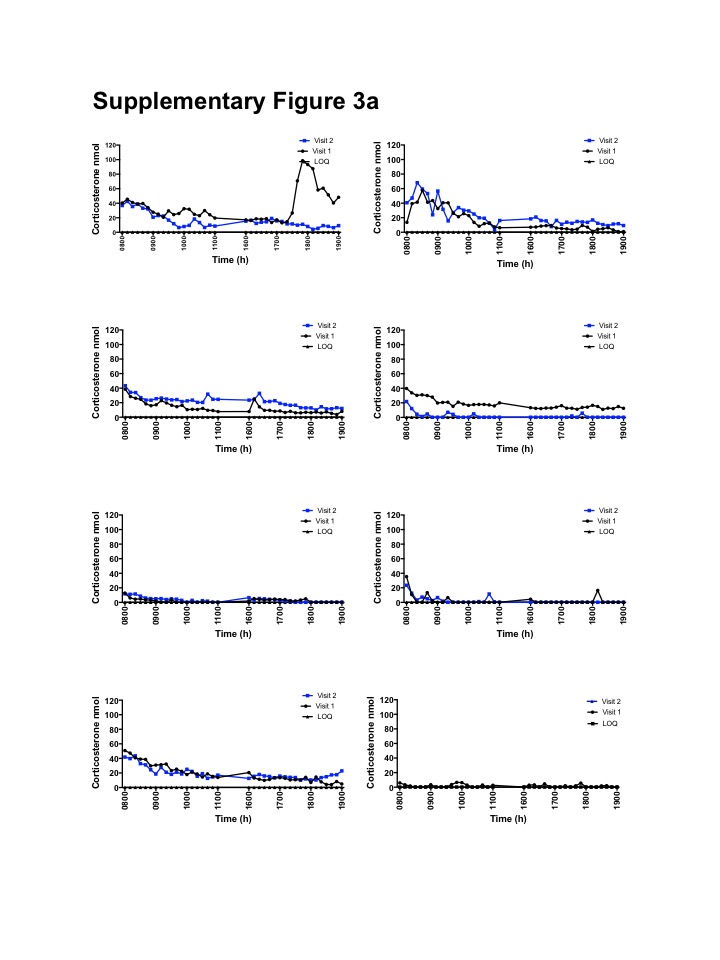

Supplement: Supplementary file 9 [file CEN-88-592-s009.jpg]

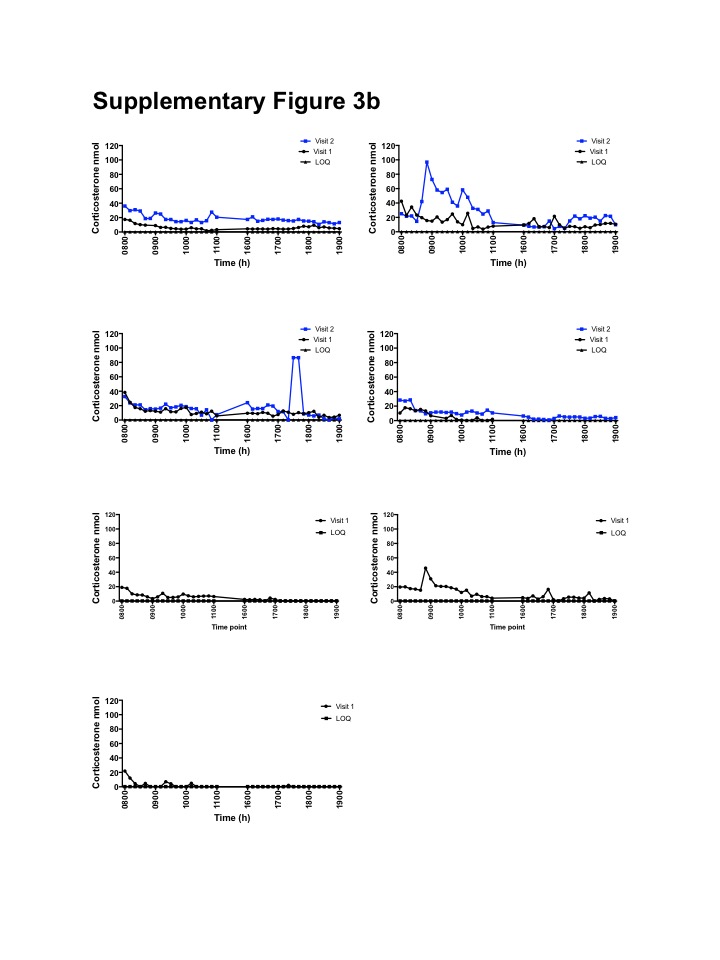

Supplement: Supplementary file 10 [file CEN-88-592-s010.jpg]

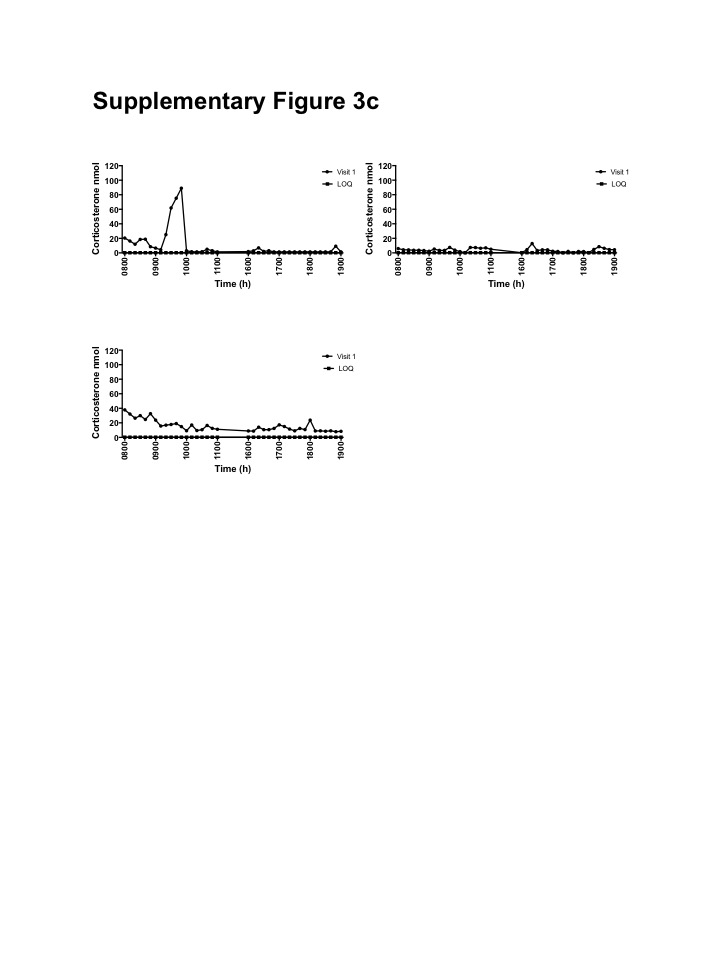

Supplement: Supplementary file 11 [file CEN-88-592-s011.jpg]

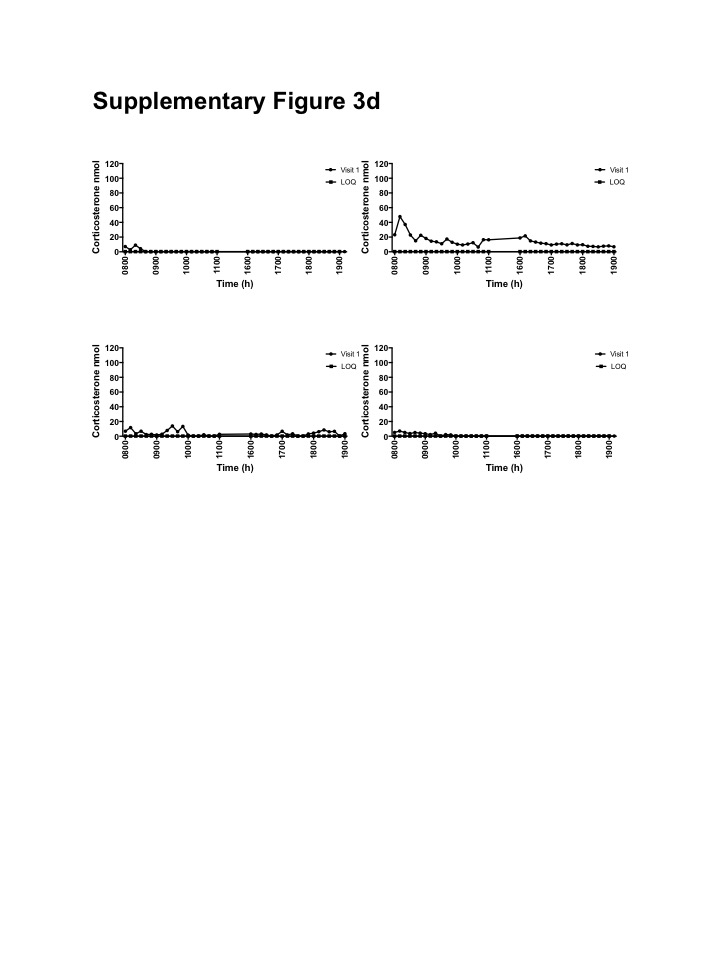

Supplement: Supplementary file 12 [file CEN-88-592-s012.jpg]

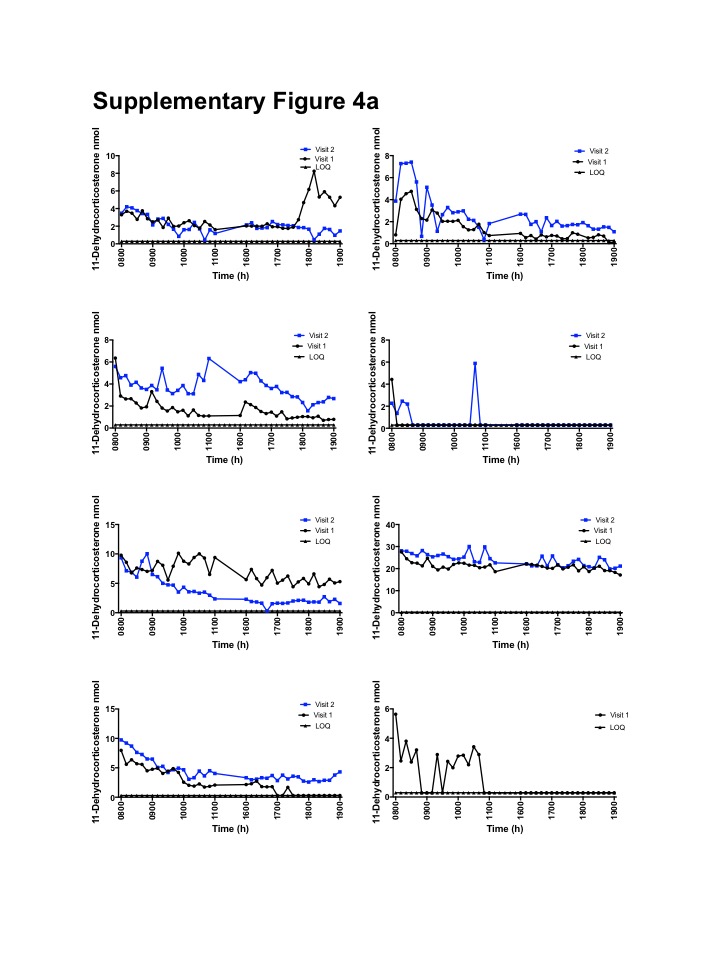

Supplement: Supplementary file 13 [file CEN-88-592-s013.jpg]

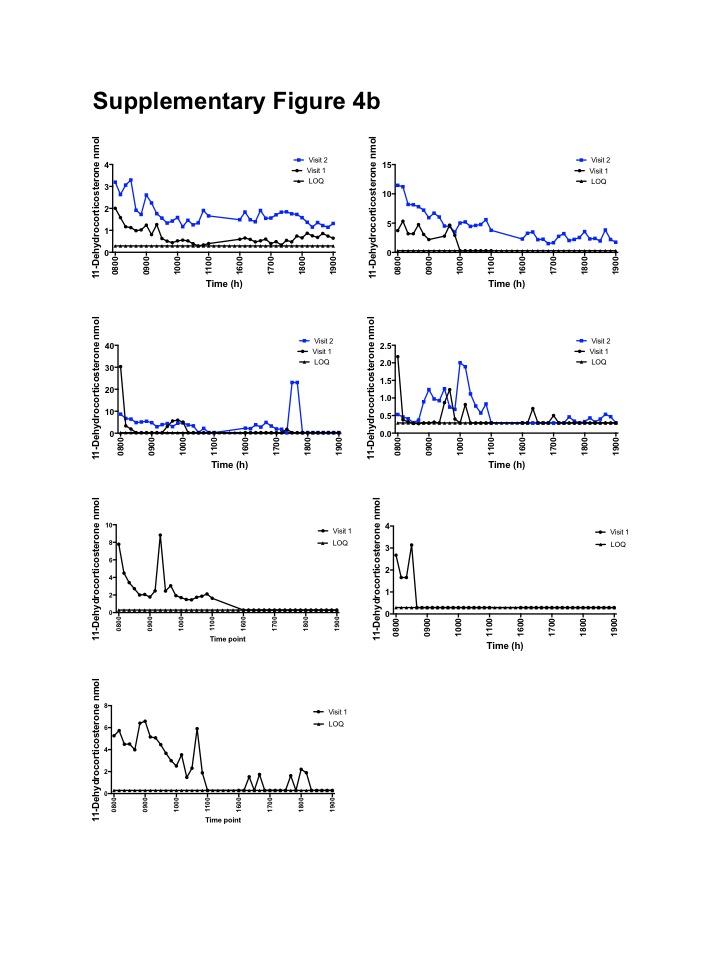

Supplement: Supplementary file 14 [file CEN-88-592-s014.jpg]

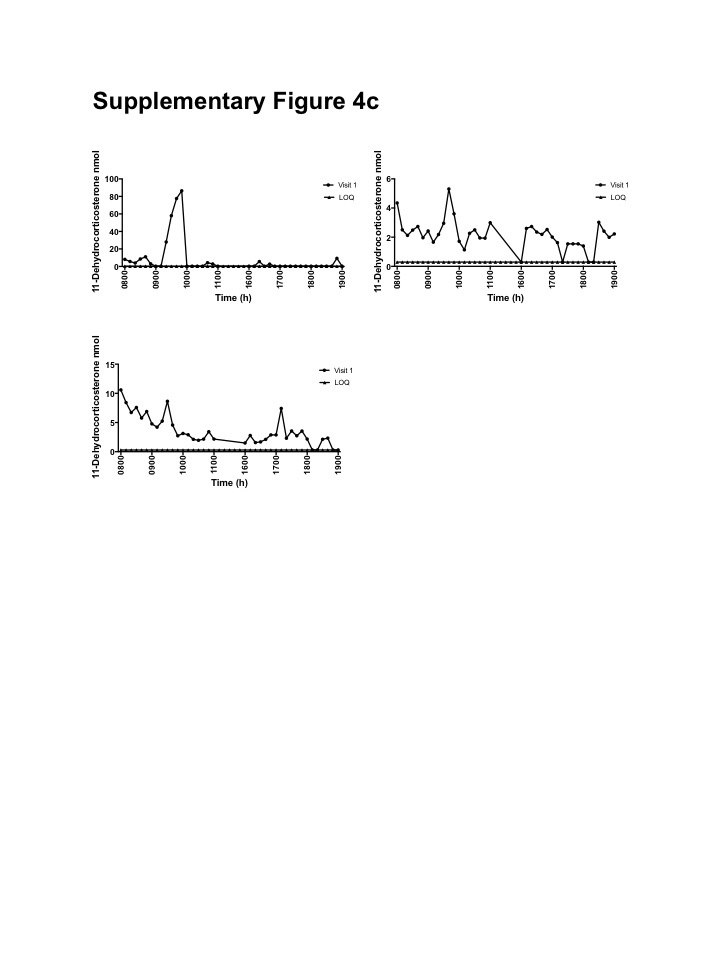

Supplement: Supplementary file 15 [file CEN-88-592-s015.jpg]

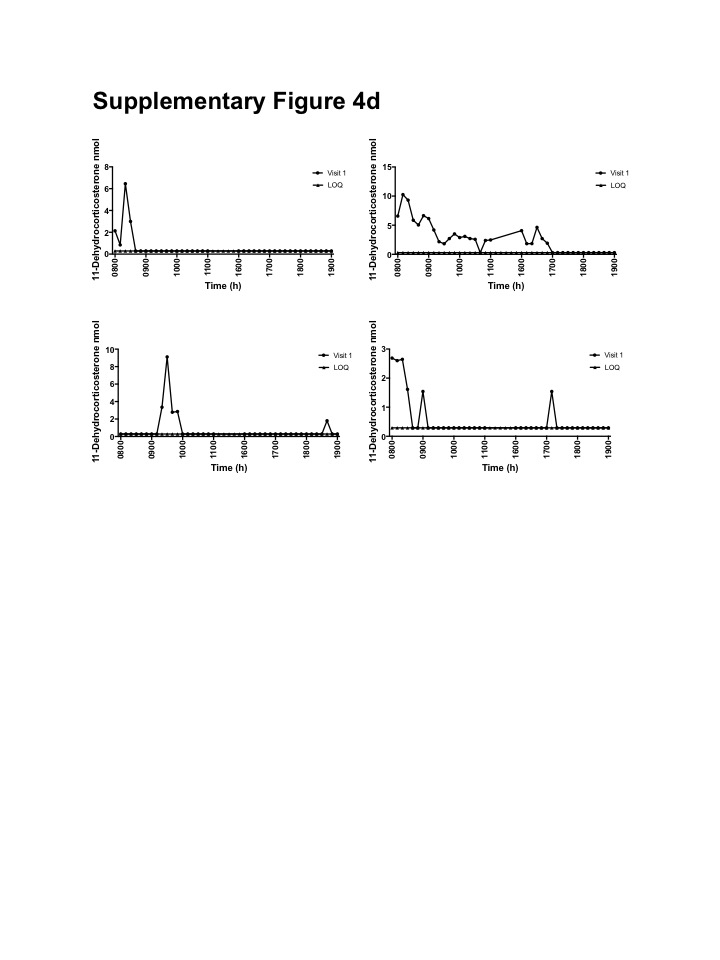

Supplement: Supplementary file 16 [file CEN-88-592-s016.jpg]
