# Supplementary material for: Pulsatility of glucocorticoid hormones in pregnancy: Changes with gestation and obesity
Source: Clin Endocrinol (Oxf). 2018 Jan 29;88(4):592–600. doi: 10.1111/cen.13548 (PMC5887976; doi:10.1111/cen.13548)
Supplement: Supplementary file 17 [file CEN-88-592-s017.docx]

**Supplementary Tables**

# Supplementary Table 1 Mass spectral conditions for analysis of analytes and internal standards by positive ion electrospray ionisation

Key : Quan, quantifier ion; Qual, qualifier ion; V, volts

|  | Molecular Weight g/mol | Precursor ion (*m*/*z*) | Product ion (m/z)  Quan; Qual | Declustering Potential  (V) | Collision energy  (V)  Quan; Qual | Cell exit potential  (V)  Quan; Qual |
| --- | --- | --- | --- | --- | --- | --- |
| **ANALYTES** | | | | | | |
| Cortisol | 362.2 | 363.2 | 121, 77 | 131 | 29, 101 | 14, 14 |
| Cortisone | 360.2 | 361.2 | 77; 163.2 | 166 | 99; 11 | 36; 20 |
| Corticosterone | 346.1 | 347.1 | 121.1; 97.1 | 66 | 69; 69 | 8; 8 |
| 11-dehydrocorticosterone | 344.1 | 345.1 | 121.2; 90.9 | 51 | 33; 71 | 8; 10 |
| **INTERNAL STANDARDS** | | | | | | |
| D_4_-cortisol | 366.5 | 367.0 | 121; only one | 121 | 25 | 20 |
| Epi-cortisol | 362.5 | 363.2 | 121; only one | 131 | 29 | 14 |
| Epi-corticosterone | 340.1 | 347.1 | 121; only one | 66 | 69 | 8 |

# Supplementary Table 2 Intra-assay and inter-assay precision and accuracy, obtained from aqueous standards as a surrogate matrix.

|  | **Target Concentration**  **(ng/200 μL)** | **Intra-assay (*n=6*)** | | | **Inter-assay (*n=6*)** | | |
| --- | --- | --- | --- | --- | --- | --- | --- |
|  |  | **Concentration (ng/200 μL): mean (SD)** | **Precision**  **(% RSD)** | **Accuracy**  **(%)** | **Concentration (ng/200 μL): mean (SD)** | **Precision**  **(% RSD)** | **Accuracy (%)** |
| **Cortisol** | Low (2.5) | 2.4 (0.2) | 9.0 | 96 | 2.4 (0.4) | 15.9 | 97 |
|  | Mid (100) | 102.1 (5.4) | 5.3 | 102 | 99.2 (9.8) | 9.8 | 99 |
|  | High (200) | 196.9 (6.0) | 3.0 | 98 | 195.3 (13.5) | 6.9 | 98 |
| **Cortisone** | Low (2.5) | 2.2 (0.2) | 11.0 | 87 | 2.7 (0.5) | 18.3 | 107 |
|  | Mid (100) | 95.9 (6.9) | 7.2 | 96 | 48.5 (3.2) | 6.6 | 97 |
|  | High (200) | 212.9 (13.7) | 6.4 | 106 | 198.4 (4.2) | 2.1 | 99 |
| **Corticosterone** | Low (0.1) | 0.1 (0.005) | 4.7 | 107 | 0.1 (0.01) | 10.7 | 100 |
|  | Mid (10) | 9.5 (0.07) | 7.0 | 95 | 10.0 (1.1) | 10.9 | 100 |
|  | High (25) | 25.1 (0.7) | 2.8 | 100 | 25.3 (1.7) | 6.6 | 101 |
| **11-dehydrocorticosterone** | Low (0.1) | 0.11 (0.0) | 14.4 | 85 | 0.10 (0.0) | 7.3 | 104 |
|  | Mid (15) | 16.1 (2.0) | 12.5 | 107 | 14.9 (1.1) | 7.5 | 100 |
|  | High (25) | 24.3 (2.6) | 10.6 | 97 | 26.1 (2.5) | 9.7 | 105 |

**Supplementary Table 3**

|  | **Hormone Characteristics** | **Group** | | | | | | **p-value** | |
| --- | --- | --- | --- | --- | --- | --- | --- | --- | --- |
|  |  | **LNP** | **LP1** | **LP2** | **ONP** | **OP1** | **OP2** | **Lean** | **Obese** |
| **Serum** | **Baseline fasting**  Cortisol | 440.9 (85.6) | 797.4 (325.2) | 892.6 (221.2) | 326.3 (112.0) | 759.9 (290.2) | 840.5 (128.8) | 0.017* | 0.027* |
|  | **Highest peak (**nmol)  Cortisol  Cortisone  Corticosterone  11-dehydrocorticosterone | 347.0 (94.5)  109.1 (40.6)  16.2 (15.1)  33.5 (46.0) | 643.1 (314.3)  118.1 (37.4)  32.1 (29.9)  7.9 (7.6) | 859.9 (266.3)  151.2 (68.1)  31.1 (11.3)  9.2 (9.3) | 215.6 (33.2)  73.4 (13.6)  8.5 (10.1)  4.9 (2.9) | 569.8 (294.5)  160.2 (200.9)  18.5 (15.4)  6.1 (3.7) | 600.9 (200.5)  166.3 (67.8)  60.5 (36.3)  13.3 (9.7) | 0.017*  0.342  0.418  0.469 | 0.013*  0.311  0.015*  0.269 |
|  | **Lowest trough (**nmol)  Cortisol  Cortisone  Corticosterone  11-dehydrocorticosterone | 136.5 (58.6)  48.7 (5.8)  3.4 (4.4)  0.3 (0.0 | 230.5 (98.8)  48.5 (11.4)  4.5 (5.1)  3.8 (6.7) | 353.9 (104.5)  68.4 (12.6)  4.7 (4.6)  3.8 (7.4) | 92.6 (28.8)  73.4 (13.6)  3.6 (2.7)  1.0 (0.8) | 343.3 (189.9)  45.9 (20.6)  2.0 (2.1)  0.7 (0.8) | 234.0 (80.4)  74.8 (12.2)  4.1 (4.7)  0.9 (0.8) | <0.0001*  0.002*  0.992  0.525 | 0.035*  0.022*  0.642  0.833 |
|  | **Profile AUC (**nmol)  Cortisol  Cortisone  Corticosterone  11-dehydrocorticosterone | 9231.8 (1681.2)  2791.8 (289.6)  419.6 (237.9)  194.4 (136.3) | 17937.1(7141.6)  3075.9 (753.7)  577.0 (456.3)  195.8 (281.3) | 24345.7 (7328.2)  4493.8 (1131.0)  603.9 (349.2)  250.1 (330.6) | 6854.9 (628.4)  1966.7 (270.0)  603.9 (349.2)  47.1 (41.4) | 14498.6 (5974.1)  2450.2 (1022.8)  297.9 (185.5)  47.2 (30.6) | 24660.1 (8937.4)  4708.9 (1124.1)  736.4 (307.0)  107.8 (73.7) | <0.0001*  0.004*  0.157  0.457 | <0.0001*  0.019*  0.081  0.564 |
| **Interstitial Fluid Cortisol** | **Pulse frequency** (pulse/hr) | 0.23 (0.09) | 0.202 (0.14) | 0.23 (0.13) | 0.22 (0.12) | 0.18 (0.12) | 0.04 (0.003) | 0.591 | 0.065 |
|  | **Pulse height** (amplitude) (ug/dL) | 0.002 (0.0007) | 0.0032 (0.002) | 0.0023 (0.002) | 0.0016 (0.002) | 0.0035 (0.002) | 0.0022 (0.002) | 0.541 | 0.478 |
|  | **Mean concentration** (ug/dL) | 0.34 (0.26) | 0.57 (0.28) | 0.57 (0.43) | 0.23 (0.18) | 0.58 (0.25) | 0.23 (0) | 0.835 | 0.111 |

Serum cortisol, cortisone, corticosterone, 11-dehydrocorticosterone in lean and obese pregnant and non-pregnant subjects. Data are mean (sd). P-values are from one-way ANOVA comparing variables in non-pregnancy, pregnancy visit 1 and pregnancy visit 2.

**Key:** LNP, Lean non-pregnant; LP1, lean pregnant visit 1; LP2 lean pregnant visit 2; ONP, obese non-pregnant; OP1, obese pregnant visit 1; OP2, obese pregnant visit 2. Significant figure represented by asterix (*).
